# Enrichment and Purification of Casein Glycomacropeptide from Whey Protein Isolate Using Supercritical Carbon Dioxide Processing and Membrane Ultrafiltration

**DOI:** 10.3390/foods3010094

**Published:** 2014-01-09

**Authors:** Laetitia M. Bonnaillie, Phoebe Qi, Edward Wickham, Peggy M. Tomasula

**Affiliations:** Dairy and Functional Foods Research Unit, Eastern Regional Research Center, Agricultural Research Service, United States Department of Agriculture, 600 East Mermaid Lane, Wyndmoor, PA 19038, USA; E-Mails: phoebe.qi@ars.usda.gov (P.Q.); edward.wickham@ars.usda.gov (E.W.)

**Keywords:** whey proteins, fractionation process, supercritical carbon dioxide, casein glycomacropeptide, β-lactoglobulin, membrane filtration

## Abstract

Whey protein concentrates (WPC) and isolates (WPI), comprised mainly of β-lactoglobulin (β-LG), α-lactalbumin (α-LA) and casein glycomacropeptide (GMP), are added to foods to boost nutritional and functional properties. Supercritical carbon dioxide (SCO_2_) has been shown to effectively fractionate WPC and WPI to obtain enriched fractions of α-LA and β-LG, thus creating new whey ingredients that exploit the properties of the individual component proteins. In this study, we used SCO_2_ to further fractionate WPI via acid precipitation of α-LA, β-LG and the minor whey proteins to obtain GMP-enriched solutions. The process was optimized and α-LA precipitation maximized at low pH and a temperature (T) ≥65 °C, where β-LG with 84% purity and GMP with 58% purity were obtained, after ultrafiltration and diafiltration to separate β-LG from the GMP solution. At 70 °C, β-LG also precipitated with α-LA, leaving a GMP-rich solution with up to 94% purity after ultrafiltration. The different protein fractions produced with the SCO_2_ process will permit the design of new foods and beverages to target specific nutritional needs.

## 1. Introduction

With the introduction of processing methods, such as microfiltration and ultrafiltration, whey protein concentrates (WPC) with protein contents ranging from 35% to 85% and whey protein isolates (WPI) with protein contents greater than 90% are available to improve the functional and nutritional properties of foods. The proteins of cheese whey comprise mainly β-lactoglobulin (β-LG, 48%–58%), α-lactalbumin (α-LA, 13%–19%), caseinomacropeptide, also called glycomacropeptide (GMP, 12%–20%), bovine serum albumin, heavy and light chain immunoglobulins and several minor whey proteins [1]. The WPC or WPI products may vary from processor-to-processor, due to differences in manufacturing protocols, the history of heat treatment and the type of cheese used to produce the whey [2]. There is less variability in the functional properties of WPI products, because they contain less fat and lactose than WPC products. For consistent food product quality, food processors often blend WPC from different sources to assure a uniform product [2].

To circumvent the problems associated with variations in the whey products, several processes have been developed [1] to fractionate the proteins found in WPC and WPI and emphasize the production of an α-LA-enriched fraction and a β-LG-enriched fraction [3,4,5]. The α-LA-enriched fraction has been proposed for use in humanized infant formulations and for foods for children and seniors [6,7,8,9], and α-LA is also high in branched chain amino acids. The β-LG enriched fraction may be used to enhance gel strength in foods, but more importantly, it is high in branched chain amino acids, leucine and other essential amino acids [10,11], which may be used to fortify foods or in the creation of foods that target specific nutritional needs.

In previous work [3,5,12,13], a process was described to produce enriched fractions of α-LA and β-LG from solutions of WPC and WPI using supercritical CO_2_ (SCO_2_) at temperatures (*T*) up to 65 °C and SCO_2_ pressures (*P*) up to 34 MPa. When SCO_2_ is dissolved in aqueous solution, carbonic acid is formed, which lowers the pH of the solution, depending on the thermodynamic equilibrium at *T* and *P* [14], as well as the concentration and buffering properties of the whey proteins. Fractions containing enriched concentration of α-LA or β-LG can be obtained by carefully controlling the operating conditions [3,4,5].

While much is known about α-LA and β-LG, interest in GMP is growing, since it is a bioactive peptide with unique nutritional and nutraceutical properties. GMP is a by-product of the cheese-making process and is obtained when chymosin causes the hydrolysis of κ-casein at the Phe^105^–Met^106^ bond with the release of GMP, residues 106 to 169 [15], and does not contain phenylalanine [11]. Thus, one of the most important potential applications of GMP is the creation of foods for phenylketonuria patients, individuals who cannot metabolize phenylalanine [16,17,18]. Many nutraceutical properties of GMP have been reported that include dental cavity prevention, appetite suppression and immune system support [19,20,21,22,23].

Many processes have been developed for large-scale production of GMP from whey and include ultrafiltration, ion-exchange chromatography, solvent partition [24,25] and complexation [1,26,27,28]. Most methods in use today employ ultrafiltration [24,29,30,31,32,33,34] or ion-exchange chromatography [18,35,36,37,38,39,40]. Variable recoveries have been reported ranging from 34% by ultrafiltration to 98% by anion-exchange chromatography [38]; however, these processes required salts, solvents, buffers and acids, which introduce contaminants into the GMP product that must be removed through additional processing.

The quantification of GMP in dairy protein fractions and products, including enzymatically-treated casein, cheese whey, WPC or WPI, is generally performed through the identification and measurement of characteristic peaks with high performance liquid chromatography (HPLC) [41,42,43,44,45,46] or capillary zone electrophoresis [47], coupled with mass spectrometry [48] or gel electrophoresis [49,50] to determine the molecular weight and identity of isolated peptides.

The purpose of the present study was to fractionate WPI to prepare several new protein products using SCO_2_: (1) an enriched fraction of α-LA or of α-LA and β-LG; and (2) a fraction enriched in β-LG and GMP or a fraction containing mostly GMP. In whey protein solutions, SCO_2_ acts both as an acid and an anti-solvent, to selectively precipitate α-LA and/or β-LG, depending the on pH, pressure, temperature, protein concentration and time [3,5], eliminating the need for salts, acids and solvents. Contrary to chromatographic or other acid-based methods, most of the CO_2_ is removed immediately after depressurization, and the whey proteins fractions produced with CO_2_ are ready to consume and are not contaminated with any chemical or mineral additives. The SCO_2_ process can be scaled-up and treats concentrated solutions; thus, commercial quantities of enriched GMP and the different whey protein fractions (depending on the processing conditions) could be produced on an industrial scale and in an environmentally-friendly way, to use as a variety of health-promoting food ingredients. The effects of several process parameters, including WPI concentration, temperature, pH, residence time and the addition of an ultrafiltration stage, on the recovery of α-LA, β-LG and GMP, and the purities of the fractions as measured with HPLC and gel electrophoresis, are presented in this manuscript.

## 2. Materials and Methods

### 2.1. Materials

Spray-dried WPI from cheese whey, Provon 190, was purchased from Glanbia Nutritionals Inc. (Richfield, ID, USA) and contained 90.1 wt% protein, with 3.6 wt% moisture, 2.9 wt% ash and, by difference, 3.4 wt% lactose and fat (manufacturer’s analysis). The protein composition as measured with gel electrophoresis (SDS-PAGE, 5 replicates) and corrected with an α-LA/β-LG calibration curve was: 67.8 wt% β-LG, 25.0 wt% α-LA and 72 wt.% caseins and minor whey proteins (immunoglobulins (Ig), bovine serum albumin (BSA), lactoferrin (Lf) and casein fragments). The measurement and calculation of GMP contents were performed using an HPLC/mass-spectrometry/total nitrogen combination method (described below), and the composition of the WPI was corrected accordingly, to approximately 18% GMP, 56% β-LG, 20.5% α-LA and 5.5% other whey proteins.

WPI solutions ranging from 20 to 100 g·L^−1^ were prepared using de-ionized water produced with a Milli-Q Synthesis water purification system (Millipore, Billerica, MA, USA). Liquid carbon dioxide (CO_2_) tanks with an eductor tube were purchased from GTS-Welco (Allentown, PA, USA).

### 2.2. Enrichment of β-LG and GMP Using Supercritical CO_2_

The pilot-scale SCO_2_ fractionation process is described in detail in prior works by Bonnaillie *et al*. [3,5]. Previously used at temperatures (*T*) below 65 °C, the process was modified to study GMP-enrichment up to 70 °C. The 1-L high-pressure batch reactor was loaded with 500 mL WPI solutions of various concentrations, *C*_WPI_ = 20–100 g·L^−1^. The reactor was heated to the desired temperature, *T* = 60–70 °C, and pressurized with liquid CO_2_ to the desired pressure, *P* = 6–34 MPa. *T* and *P* were monitored during start-up and steady-state, throughout the duration of the experiment (residence time, *t*).

The pH of the WPI solution under pressure was calculated from *P* and *C*_WPI_ using the model for WPI solutions saturated with SCO_2_ of Bonnaillie and Tomasula [5]. After mixing with SCO_2_ for 1–4 h at *T* and *P*, the WPI mixture was cooled to ~40 °C, then extracted. The WPI mixture foamed during extraction, due to vaporization of the dissolved CO_2_ when reaching atmospheric pressure; after foam collapse, the pH of the sample was ~6.0, and the mixture was collected in a 750-mL centrifuge bottle.

### 2.3. Separation and Quantification of the Protein Fractions

Samples were centrifuged in a large capacity refrigerated bench top centrifuge with bucket holders (Fisher Scientific, Pittsburg, PA, USA) at 4000× *g* for 60 min. The supernatant, or soluble fraction, was named the “beta” (β) fraction, that is the protein fraction enriched with β-LG; the aggregated fraction was lyophilized and named the “alpha” (α) fraction, that is the protein fraction enriched with α-LA. Both protein fractions were quantified by mass difference: before centrifugation, after removal of the supernatant and after lyophilization.

### 2.4. Ultrafiltration of Soluble Fractions

Ultrafiltration to separate GMP from β-LG was performed with a 400-mL Amicon stirred cell 8400, equipped with Ultracel regenerated cellulose membranes of sizes of 10 or 30 kDa (kg·mol^−1^) (all Millipore, Billerica, MA, USA). The membranes were pre-conditioned before use according to manufacturer instructions. Aliquots (50 or 100 mL volumes) of protein solutions (β fractions) were filtered under pressurized nitrogen (0.21 MPa) for 120 min, then two washes were performed successively by the addition of the same volume of deionized water and filtered for 120 min. each to help collect GMP in the filtrate. Samples of the filtrate were taken at the end of each stage for HPLC analysis. The final retentate and filtrate fractions were collected, measured and lyophilized for further analysis.

### 2.5. Compositional Analysis of the Enriched Protein Fraction

The proportions of α-LA, minor whey proteins and β-LG remaining in the soluble fraction were determined using sodium-dodecyl-sulfate polyacrylamide gel electrophoresis (SDS-PAGE) on a Phast System (Pharmacia, Piscataway, NJ, USA) with Phast homogeneous gels containing 20% acrylamide and 8 lanes. Samples were prepared similarly to the method described by Parris *et al*. [51] and adapted by Bonnaillie *et al*. [3,5]. A calibrated α-LA/β-LG mixture containing 20 wt% α-LA and 80 wt% β-LG standards was used in two of the gel lanes. The composition of the protein solution calculated by the ImageQuaNT™ software (Molecular Dynamics Inc., Sunnyvale, CA, USA) was corrected according to the calibration sample.

Because GMP is not visible on SDS-PAGE gels, the soluble fractions were also analyzed using reversed phase high-pressure liquid chromatography (RP-HPLC), using the method of Bonnaillie and Tomasula [5]. Gradient elution was carried out with a mixture of two solvents: eluant A contained 100% acetonitrile and 0.1% trifluoroacetic acid (TFA), and eluant B contained 100% water and 0.1% TFA. The elution gradient was set as follows: 0 min: 30% A; 0–30 min: 30%–50% A; 30–40 min: 50%–90% A; with 5 min column re-equilibration at 90% A. The flow rate was 0.8 mL·min^−1^, and the column temperature was maintained at 30 °C. UV-absorbance was monitored at 214 nm.

Mass spectrometry was used to identify the molecular weight of the compounds corresponding to the different peaks during an HPLC run. The collected compounds were analyzed with a 4700 Proteomics Analyzer with Matrix-assisted laser desorption/ionization with automated tandem time of flight fragmentation of selected ions mass spectrometer (MALDI-TOF/TOF, Applied Biosystems, Framingham, MA, USA) in the positive linear mode. Spectra were obtained by averaging 1000 acquired spectra in the MS mode. The conversion of TOF to molecular weight for the protonated protein, [M + H]^+^, was based on the calibration of the instrument with a protein standard calibration kit (Applied Biosystems).

Total solids content, *C*_solids_, of the soluble fractions was obtained by the mass difference of 5-mL liquid samples after drying in an oven at 105 °C. The total protein content, *X*_protein_, of the soluble fractions was measured using the AOAC International (Association of Official Agricultural Chemists) official method 991.20, the Kjeldahl method for total nitrogen content of milk proteins with a factor of 6.38 [52]. Ash content was measured using the AOAC official method 945.46, calculating the mass difference for 0.6 g of lyophilized protein sample placed overnight in the furnace at 550 °C. The lactose content of lyophilized protein samples was measured with a YSI 2700 Select Biochemistry Analyzer (YSI Inc. Life Sciences, Yellow Springs, OH, USA) using Application Note No. 320, “Lactose measurement in cheese”. Compositional analyses were performed in triplicate.

## 3. Results and Discussion

### 3.1. Fractionation of WPI with SCO_2_

**Figure 1:**
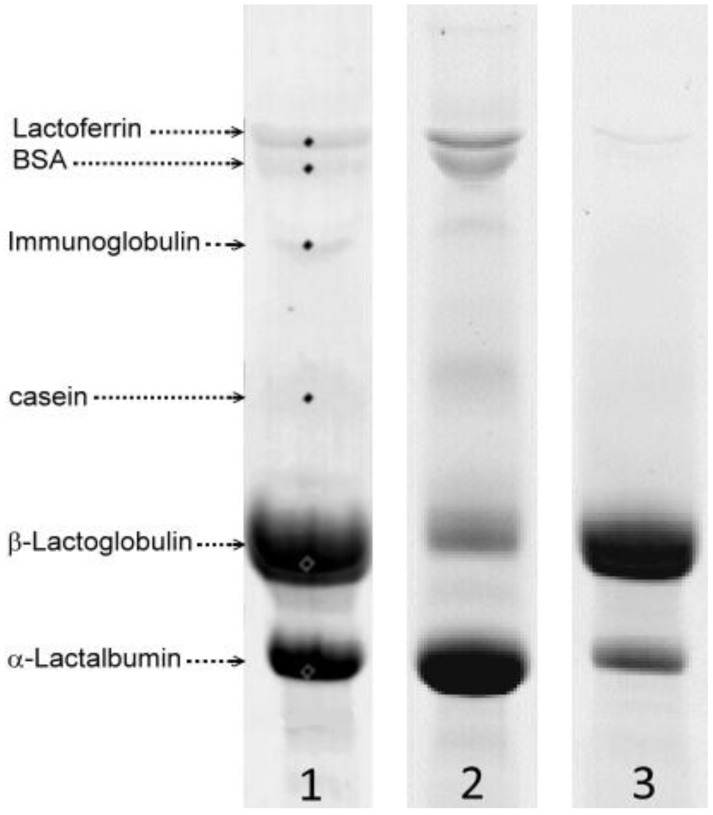
Typical distribution of the whey proteins before and after processing with SCO_2_ as measured with SDS-PAGE: *1*, Initial whey protein isolates (WPI); *2*, precipitate (alpha) fraction; *3*, soluble (β) fraction. BSA, bovine serum albumin.

The supercritical CO_2_ reactor effectively enabled fractionation of the proteins of WPI into a solid, α-LA-enriched fraction and a soluble, β-LG-enriched fraction, via precipitation of α-LA under acidic conditions at 60 to 70 °C and under CO_2_ pressures of 6–34 MPa for up to 4 h. As demonstrated in previous work, neither fraction contained salt, acid or any other contaminant after processing and had a final pH of 6.0. Figure 1 shows the typical protein distribution of the two enriched fractions as measured with SDS-PAGE and compared with the WPI feed.

The starting WPI contained almost three times more β-LG than α-LA, and a small amount of lactoferrin (Lf), bovine serum albumin (BSA), immunoglobulin (Ig) and residual caseins (lane 1). After SCO_2_ processing, the precipitated fraction was typically comprised of mainly α-LA and most of the minor whey proteins, with a non-negligible amount of co-precipitated or entrapped β-LG (lane 2). On the other hand, the soluble fraction contained mostly β-LG, some remaining non-precipitated α-LA and little to no minor whey proteins (lane 3). The total amount of precipitated proteins and the relative proportions of all proteins in the two fractions greatly depended on the process parameters, namely starting concentration (*C*_WPI_), temperature (*T*), pH (a function of CO_2_ pressure, *P*) and residence time (*t*), which influence the aggregation kinetics of the different whey proteins, as described by Bonnaillie and others [3,4,5].

**Figure 2:**
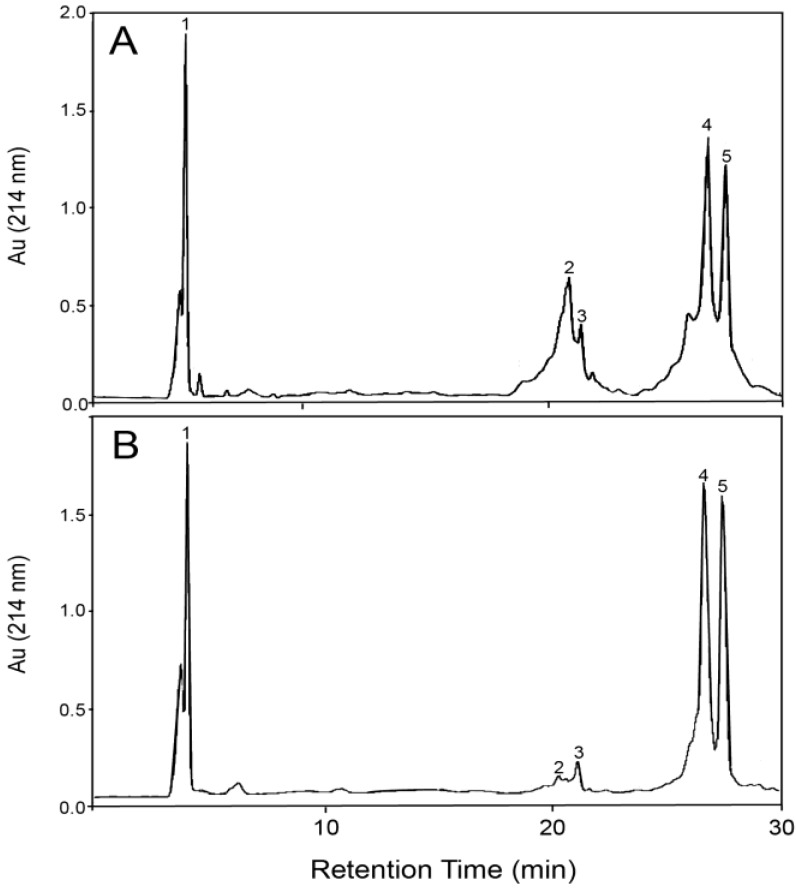
Reversed-phase (RP)-HPLC chromatograms of whey protein mixtures: (**A**) starting WPI; (**B**) soluble (β) fraction after supercritical carbon dioxide (SCO_2_) processing. Peak assignments: 1, glycomacropeptide (GMP) (3.7–4.0 min); 2,3, α-lactalbumin (α-LA) (19.2–20.6 min); 4,5, β-lactoglobulin (β-LG) (25–27 min).

**Figure 3:**
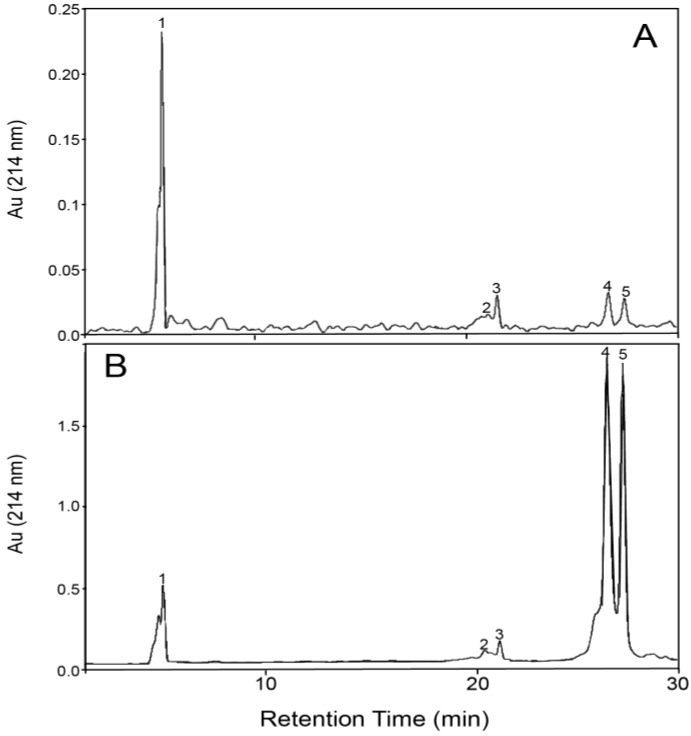
RP-HPLC chromatograms: (**A**) enriched GMP fraction (filtrate, 58% GMP) and (**B**) enriched β-LG fraction (retentate, 84% β-LG) after treatment of a 10% WPI solution with SCO_2_ at 65 °C and pH 4.6, ultrafiltration with 30-kDa membrane and two washes. Peaks assignment: 1, GMP (3.7–4.0 min); 2,3, α-LA (19.2–20.6 min); 4,5, β-LG (25–27 min).

The presence of large quantities of GMP in both the starting WPI and the soluble fraction after processing was identified with reversed-phase HPLC (RP-HPLC) (Figure 2). The peaks for α-LA appeared at retention times between 19.2 and 20.6 min (peaks 2 and 3), while the peaks corresponding to β-LG typically appeared between 25 and 27 min (peaks 4 and 5), as confirmed with α-LA and β-LG standards. All other minor peaks between 4.5 and 30 min were attributed to BSA, Ig, Lf and caseins, according to their standards, while the group of peaks between 3.7 and 4.0 min retention time (peak 1) was identified with mass-spectrometry as belonging to a macropeptide of an average molecular weight of 6780 g·mol^−1^, the characteristic molecular weight of glycomacropeptide (GMP) [44]. The shapes, sizes and relative positions of the different groups of peaks closely matched those described by Thoma *et al*. [46] in their detailed study of the RP-HPLC analysis of whey protein mixtures containing GMP, where GMP was separated between its different non-glycosylated (CMP) and glycosylated (GMP) variants. In Figure 2 and Figure 3, the variants of CMP/GMP were grouped together under peak 1.

The differences between Figure 2A and Figure 2B demonstrate the effect of the SCO_2_ fractionation process on the composition of the WPI solution: with time, acidity and heat having caused the precipitation of most of the α-LA proteins, as the corresponding peaks for α-LA at retention times 19.2–20.6 min are greatly reduced on Figure 2B compared to Figure 2A. In addition, the reduction or disappearance of almost all other small intermediate peaks corresponding to the minor whey proteins demonstrates their co-precipitation with α-LA in the aggregate fraction. Consequently, the peaks corresponding to GMP (peak 1) and β-LG (peaks 4 and 5) became more pronounced. Two clearly distinct β-LG peaks were observed due to the two β-LG genetic variants, with peak 4 being genetic variant A and peak 5 being genetic variant B. The overall chromatogram (Figure 2B) approached that of a binary β-LG/GMP protein system.

Table 1 lists the compositional analysis of a selection of soluble beta fractions as a function of SCO_2_ processing parameters (*C*_WPI_, *T*, calculated pH and *t*), compared with the starting (untreated) WPI, as measured with HPLC and compared with the SDS-PAGE data. Some samples produced during a prior study [5] (rows 1, 3, 9 and 10) were reanalyzed and recalculated to obtain precise GMP contents and recovery rates.

For each sample, the HPLC peak areas (as in Figure 2B) were integrated for α-LA and β-LG and converted to concentrations *C*_A_ and *C*_B_, respectively, using the peak areas of the standard α-LA/β-LG protein mixture measured during each calibration run. The approximate total concentration of all other whey proteins and peptides, mainly the GMP and other minor whey proteins, *C*_other_, was calculated with:

*C*_other_ = *C*_solids_ × *X*_protein_ − *C*_A_ − *C*_B_ (1)

All the samples analyzed with the Kjeldahl method had a total protein content close to 80%; thus it was reasonable to use this value as a good approximation of *X*_protein_ for the samples that were not subjected to nitrogen content analysis.

**Table 1:** HPLC analysis of the compositions of selected soluble (β) fractions after SCO_2_ treatment of WPI as a function of the process parameters.

No.	*T* (°C)	*C*_WPI_ (g·L^−1^)	pH	*t* (min)	*C*_solids _(g·L^−1^)	*X*_protein_ (wt%)	*C*_A_ (g·L^−1^)	*C*_B_ (g·L^−1^)	*C*_other_ (g·L^−1^)	*C*_GMP_ (g·L^−1^)	Recovery		HPLC Composition			Ratio β-LG/α-LA	
											recA (%)	recB (%)	α-LA (wt%)	β-LG (wt%)	GMP (wt%)	HPLC	SDS-PAGE
0.	*WPI Feed*			-	-	-	18.3	50.1	-	16.1	-	-	20.3	55.7	17.9	2.74	2.71
1.	60	100	5.0	215	85.3	80	6.6	45.1	16.6	14.9	36.1	90.0	9.9	67.8	22.3	6.8	7.1
2.		100	4.9	185	85.7	80.9	6.6	45.7	17.0	14.6	36.1	91.2	9.9	68.3	21.8	6.9	7.8
3.		70	4.6	155	51.6	80	4.8	26.9	9.5	8.7	37.4	76.7	11.8	66.7	21.5	5.7	5.7
4.		20	4.2	120	18.9	83.6	3.0	9.2	3.6	3.5	82.0	91.8	19.1	58.8	22.1	3.1	2.6
5.	62	100	5.0	180	81.6	81.1	6	49.2	11.0	14.4	32.8	98.2	8.6	70.7	20.7	8.2	8.5
6.		100	4.7	310	81.5	80	3.6	43.2	18.4	13.5	19.7	86.2	6.0	71.6	22.4	12.0	10.2
7.		100	4.6	120	77.0	80	5.9	42.8	12.9	13.8	32.2	85.4	9.4	68.5	22.1	7.3	7.0
8.		70	4.6	180	48.8	80	3.7	25.1	10.2	9.2	28.9	71.6	9.7	66.1	24.2	6.8	5.7
9.	65	100	5.0	160	75.1	78.3	2.4	36.9	19.5	13.0	13.1	73.6	4.6	70.6	24.8	15.4	16.3
10.		100	4.6	140	58.7	79	1.8	24.6	20.0	12.2	9.8	49.1	4.7	63.6	31.7	13.6	9.5
11.	70	100	4.9	305	43.6	80	3.0	6.6	*-*	13.6	16.4	13.2	13.0	28.3	58.7	2.2	2.0

*T* = fractionation temperature in the reactor; *C*_WPI_ = initial WPI Concentration; *t* = residence time in the reactor; α-LA = α-lactalbumin; β-LG = β-lactoglobulin; GMP = casein glycomacropeptide. Concentrations and compositions in the soluble fraction: *C*_solids_ = total solids concentration; *X*_protein_ = mass fraction of proteins in the solids; *C*_A_ = α-LA concentration; *C*_B_ = β-LG concentration; *C*_other_ = total concentration of all other proteins; *C*_GMP_ = GMP concentration.

To calculate the concentration of GMP in each sample, the extinction coefficient of GMP at 214 nm was assumed to be equal to that of β-LG, and *C*_GMP_ was calculated using the measured peak areas for GMP, calibrated via the peak areas and concentration of the β-LG standard. Comparison of *C*_GMP_ and *C*_other_ throughout Table 1 indicates that this is a reasonable assumption, since the values of *C*_GMP_ and *C*_other_ are satisfactorily close, corresponding to the fact that most of the minor whey proteins precipitated out of the soluble fraction along with α-LA, leading to *C*_GMP_ ≈ *C*_other_, in theory. Large discrepancies between *C*_GMP_ and *C*_other_ were attributed to data scattering for *C*_other_ mostly, caused by the accumulated experimental error while measuring the different concentrations in Equation (1), due to the combination of the different analytical techniques and approximations employed.

The composition of each sample as measured with HPLC was obtained from the calculated values of *C*_A_, *C*_B_ and *C*_GMP_. The results of the HPLC analysis were further validated by comparing the ratios of β-LG to α-LA of each sample, “ratio β/α”, calculated from the HPLC data, to that obtained from independent SDS-PAGE measurement (Table 1).

The value of the ratio β/α is a useful indicator of the level of enrichment of the soluble protein fraction with β-LG compared to the initial WPI. The ratio β/α for the starting WPI had a value of 2.7, and the β-LG-enrichment process described in this work increased this value.

Generally, at constant fractionation temperature and pH, a higher starting WPI solution concentration accelerated the precipitation kinetics of α-LA without increasing β-LG precipitation greatly [4,5], producing a soluble fraction containing enriched β-LG and GMP and a lower α-LA concentration (Table 1). Increasing the fractionation temperature at constant pH and WPI concentration considerably accelerated the precipitation of α-LA and reduced the concentration (*C*_A_) and yield (recA) of α-LA in the soluble fraction, producing a soluble fraction with improved β-LG or GMP purity (e.g., rows 1, 5 and 9; 2 and 11; 3 and 8). The aggregation rate of α-LA was maximized at *T* ≥ 65 °C, which is above the thermal denaturation temperature of 63.7 °C for α-LA [53]; the precipitation of β-LG out of solution also increased significantly above 65 °C, leading to lower β-LG concentrations in the soluble fractions and, therefore, greater GMP purities (rows 9, 10 and 11). Longer residence times (*t*) and lower pH both positively influenced the amount of precipitated α-LA, that is, the removal of α-LA form the β-LG/GMP soluble fraction. The precipitation of β-LG was also found to increase with increased residence time and reduced pH, but at a slower rate than α-LA [5], because of its higher thermal denaturation temperature [54].

Mass balance on GMP for all the samples in Table 1 showed that the recovery percentage of GMP in the soluble fraction was approximately equal to the volume ratio of supernatant (the liquid fraction) to the total sample volume after centrifugation of the processed WPI suspensions. This means that the amount of GMP lost in the aggregate fraction was proportional to the volume of the wet aggregated fraction after centrifugation and that entrapment of GMP within the aggregated fraction was most likely caused by water-holding only. Therefore, GMP remained completely soluble throughout processing in the range of temperatures, CO_2_ pressures and pH values studied.

### 3.2. Fractionation of β-LG and GMP with Membrane Filtration

β-LG/GMP solutions containing the highest proportions of GMP were submitted to further treatment to simultaneously produce both a purer β-LG fraction and a purer GMP fraction, in order to leverage the different properties of the protein and macropeptide as new valuable food ingredients. Ultrafiltration technology was chosen due to the considerable size difference between GMP and β-LG at pH 6.0. β-LG typically forms non-covalent dimers with a molecular weight of ~36 kg·mol^−1^ between pH 5.2 and 8.0 [55], while the molecular weight of GMP measured via mass spectrometry in this work was 6.8 kg·mol^−1^. Accordingly, ultrafiltration membranes with 10 and 30 kg·mol^−1^ pore sizes were selected with the goal of collecting GMP in the filtrate and to retain β-LG in the retentate. Preliminary runs with the same β-LG/GMP samples and both membranes sizes showed that the 10 kg·mol^−1^ pore size effectively prevented both β-LG (36 kg·mol^−1^ size dimers) and the α-LA remnant (14 kg·mol^−1^ size soluble monomers) from passing through the membrane, producing a GMP fraction with a high purity, but also hindering the passage of GMP and restricting GMP recovery to ~25% only. On the other hand, 30 kg·mol^−1^ membranes allowed most of the GMP to pass through, while effectively retaining most, but not all, of the β-LG proteins and permitting the passage of a considerable portion of the remaining α-LA proteins. To maximize GMP recovery in the filtrate, multiple washes of the retentate with deionized water were employed, and the effects on the composition of both fractions and GMP recovery in the filtrate were examined.

Table 2 lists the operating conditions during the various ultrafiltration stages and the results of HPLC analyses of the filtrate after each stage and the final retentate for two selected β-LG/GMP solutions with a high level of GMP obtained with the SCO_2_ process under two different sets of conditions: (1) 10% WPI solution fractionated at *T* = 65 °C, pH 4.6 for 140 min, containing 31.7% GMP after centrifugation; and (2) 10% WPI solution fractionated at *T* = 70 °C, pH 4.9 for 305 min, containing 58.7% GMP after centrifugation.

Table 2 lists the volume of the retentate and total volume of the filtrate at the end of each filtration stage, the areas of the GMP and β-LG peaks as measured with RP-HPLC and the dilution used for each sample injection, the relative proportion of GMP to β-LG (w/w), the recovery yield of GMP after ultrafiltration compared to the initial amount of GMP in the sample before ultrafiltration and the purity of the GMP filtrate calculated from RP-HPLC data.

The 30-kDa membrane was effective at separating GMP from β-LG. After filtration, the β-LG content in each filtrate was greatly reduced and the GMP purity increased, with a final GMP:β-LG ratio up to 30 times that of the initial samples (from ~0.5 to ~3.5 in the first example and from ~2 to ~60 in the second example). The first and second washes were effective at increasing the amount of GMP recovered in the filtrate. However, with each wash the amount of α-LA and β-LG that passed through the membrane also increased, and the purity of the GMP fraction either remained constant or decreased. After ultrafiltration of a 10% WPI solution processed with SCO_2_ at 65 °C and pH 4.6 (first example, Table 2), the filtrate contained approximately 58% GMP with a ~60% recovery after two washes, which is approximately twice as enriched compared to the initial composition of 31.7%. On the other hand, the β-LG-enriched retentate contained 83.9% β-LG, 11.2% GMP, 3.0% α-LA and 1.9% other whey proteins. Figure 3 presents the HPLC charts of the enriched GMP fraction (Figure 3A) and the purified β-LG fraction (Figure 3B) after ultrafiltration, including two washes.

**Table 2:** Filtration and HPLC data during ultrafiltration of β-LG and GMP from selected soluble fractions produced by SCO_2_ treatment of 10% WPI solutions at 65 °C, pH 4.6, and 70 °C, pH 4.9.

Starting Composition	Membrane Pore Size (kDa)	Ultrafiltration				RP-HPLC Analysis				GMP Recovery (%)	GMP Purity (%)
		Filtration Stage	Volume Solution (mL)	Volume Filtrate (mL)	Volume Retentate (mL)	Dilution Factor	GMP Peak Area (×10^−6^)	β-LG Peak Area (×10^−6^)	Ratio GMP:β-LG		
31.7% GMP 63.6% β-LG 4.7% α-LA (65 °C, pH 4.6)	-	Initial	-	-	-	80	39.2	78.6	0.50	*-*	31.7
	10	Filtration	50	30	20	80	16.5	~0	-	25	-
	30	Filtration	50	34	16	80	25.0	6.0	4.19	43	59.7
		1st Wash	50	85	15	40	20.3	3.7	5.42	44	57.8
		2nd Wash	50	138	12	20	33.7	9.5	3.53	59	58.1
		Retentate			12	40	117.3	880.6	0.13	36	11.2
58.7% GMP 28.3% β-LG 13.0% α-LA (70 °C, pH 4.9)	-	Initial	-	-	-	80	40.7	19.6	2.07	-	58.7
	30	Filtration	100	85	15	40	43.8	~0	-	46	94.0
		1st Wash	100	189	11	20	64.2	1.2	53.8	75	87.0
		2nd Wash	100	294	6	10	90.9	1.5	59.4	82	80.4
		Retentate			6	100	73.9	163.3	0.45	14	27.1

α-LA = α-lactalbumin; β-LG = β-lactoglobulin; GMP = casein glycomacropeptide.

After ultrafiltration of a 10% WPI solution processed with SCO_2_ at 70 °C and pH 4.9, the filtrate contained as much as 94% GMP with a 46% recovery rate (no wash) or 80.4% GMP with 82% recovery after two washes, together with a β-LG-enriched retentate containing ~59.9% β-LG, 27.1% GMP and 13.0% α-LA in the proteins. The β-LG fraction was twice as enriched compared to the initial composition of 28.3%.

In all cases, the α-LA present in the initial sample partially passed through the membrane and was distributed between both the β-LG (retentate) and GMP (filtrate) fractions.

Small GMP fractions were collected during the HPLC runs and characterized with mass spectrometry to confirm the identity of the purified GMP. As expected, the molecular weight of the main macropeptide was determined to be 6780 g·mol^−1^ for all GMP fractions. In addition, the existence of a number of smaller peptides with molecular masses of 3.4 kg·mol^−1^ and lower, with unknown quantities, was also suggested by mass spectrometry, but did not appear on the HPLC chromatograms. Further study involving different analytical techniques and procedures may be employed in order to identify and quantify these smaller peptides.

Most of the lactose and ash passed through the membrane during filtration and were found in the GMP solution. Both ash and lactose comprised approximately 6% of the dry weight of the GMP fractions, each. To obtain purer GMP fractions as desired, most of the lactose and ash could theoretically be removed using an additional ultrafiltration stage with a membrane pore size of 5 kDa or lower to prevent the passage of the GMP, since GMP has a molecular weight of 6.8 kDa.

## 4. Conclusions

Supercritical CO_2_ can be used as a new green technology to enrich or isolate casein glycomacropeptide from concentrated whey protein solutions at temperatures of 65 °C or higher. After ultrafiltration, GMP purities of 94% of the proteins or higher, and enriched, soluble β-LG can be produced. The processing temperature, pressure and residence time of the SCO_2_ process, as well as the pore size of the ultrafiltration membrane, and the number of washes, can be modified to optimize the purities and yields of the GMP isolate and enriched α-LA and β-LG. All the products are free from added salts or other contaminants and ready to use as new health-promoting food ingredients.
